# Comparative in vitro study of the cleaning efficacy of AirFloss ultra and I-Prox Sulcus brushes in an orthodontic phantom model

**DOI:** 10.1038/s41598-021-81603-y

**Published:** 2021-01-21

**Authors:** Hanna Boes, Sören Brüstle, Gholamreza Danesh, Stefan Zimmer, Mozhgan Bizhang

**Affiliations:** 1 Department of Operative and Preventive Dentistry, Faculty of Health, Witten/Herdecke University, Alfred-Herrhausen-Str. 50, 58455 Witten, Germany; 2 Department of Orthodontics, Faculty of Health, Witten/Herdecke University, Witten, Germany

**Keywords:** Plaque, Oral hygiene, Fixed appliances, Preventive dentistry

## Abstract

Preventing biofilm-related risks such as gingivitis and white spot lesions during orthodontic treatments is very challenging. The cleaning efficiencies of AirFloss Ultra and I-Prox P sulcus brushes were evaluated using an orthodontic phantom model. After attaching brackets onto black-coated maxillary KaVo teeth, a plaque substitute was applied. The evaluated tooth surfaces were divided into two areas. Cleaning was performed with an AirFloss Ultra with two (A-2) or four (A-4) sprays or an I-Prox P for two (I-2) or four (I-4) seconds. Images before and after cleaning were digitally subtracted, and the percentage of fully cleaned surfaces was determined (Adobe Photoshop CS5, ImageJ). Statistical analysis was performed by ANOVA and post hoc tests with Bonferroni correction (SPSS 25, p < 0.05). The mean values of total cleaning efficacy were 26.87% for I-2, 43.73% for I-4, 34.93%, for A-2 and 56.78% for A-4. The efficacy was significantly higher for A-4 than for A-2, I-4, and I-2. There were significant differences between the four groups. Repeated cleaning led to an improved result. Within the study limitations, the AirFloss Ultra with four sprays proved to be more efficient than the sulcus brush I-Prox P for cleaning.

## Introduction

Effective oral hygiene plays a key role in preventing caries and periodontal diseases during orthodontic treatment. The attachment of brackets, bands, and arches creates additional surfaces that differ from the oral tissue structures. These retention sites provide ideal conditions for an increased accumulation of food residue and bacterial plaque by also limiting the natural cleansing mechanisms of saliva and the oral muscles^1–3^. In addition, the physiological germination pattern changes.

The presence of *Streptococcus mutans* and *Lactobacillus* represents an increased risk for the development of carious lesions^4–6^. The study of Corbett, Brown et al. showed there was an increased prevalence of *S. mutans* during orthodontic treatment. Before treatment, patients showed a lower concentration of this bacteria in the plaque than patients during treatment^7^. The concentration of *S. mutans* increased fourfold during orthodontic treatment than before treatment and decreased after treatment to the initial value^8^. As a result, initial demineralization of the enamel can be observed in this group of patients within a very short time after insertion of the orthodontic device^9^.

The incidence of developing white spot lesions during orthodontic treatment varies between 2 and 96%, and not every patient is affected similarly^3,10–13^. To avoid these side effects, preventive procedures need to be performed to reduce the proliferation of plaques and to efficiently remove them. In addition to proper nutrition and the use of fluoride, dental and oral hygiene are important.

The studies published thus far have mainly compared the effectiveness of different toothbrushes for plaque removal in multibracket patients and have reported different results^14–18^. Rosema, Slot et al. showed that the use of a toothbrush reduces plaque by 46%^19^. In particular, the hard-to-reach circular surfaces around brackets and proximal areas are not cleaned sufficiently with a toothbrush, leading to the demineralization of the enamel and periodontal diseases^20^.

Alternative cleaning procedures are therefore necessary to make the cleaning process more efficient for patients with fixed orthodontic appliances. The literature recommends using proximal cleaning devices to remove plaque in these barely accessible areas^21,22^. However, studies reviewing these proximal cleaning procedures usually consider only participants without fixed orthodontic appliances^23,24^.

In 2015, Philips presented the Sonicare AirFloss Ultra device, promising efficient plaque removal of approximal areas (the tooth surface facing an adjacent tooth) and easy use. Previous studies have shown meaningful results when applying the AirFloss^25,26^. Another possibility is to use a mono-tuft brush^27^. The v-shaped Miradent mono-tuft brush ensures gentle and efficient cleaning of hard to access areas. Both devices have the advantage that no proximal contact point needs to be bypassed during cleaning. This eases cleaning tremendously. One study showed that an oral hygiene procedure with an electric toothbrush combined with AirFloss was more effective than a manual toothbrush combined with flossing in reducing plaque and gingivitis in orthodontic subjects following 3 weeks of use^28^.

A very important parameter is the compliance of the patient^11^. A previous study proved the high acceptance and easy handling of the AirFloss. Compared to the use of dental floss, 78% of the patients preferred the AirFloss^29^. To date, no studies have described the cleaning abilities of the I-Prox P brush. However, it can be assumed that accidental sliding of the brush on the gingiva might lead to pain and therefore reduce patient compliance.

To date, no studies have published the results of the cleaning efficiency of the AirFloss Ultra compared to that of the I-Prox P brush in multibracket patients using each a single time. Thus, the aim of this prospective in vitro study was to investigate the cleaning efficiency of the AirFloss Ultra and the I-Prox P brushes on an orthodontic phantom model under standardized conditions.

## Materials and methods

The cleaning process was performed on a maxillary (upper jaw) model with full dentition of plastic teeth (KaVo Dental GmbH, Ulm, Germany).

### Preparation

First, the buccal (tooth area facing towards the cheek) and the approximal surfaces of the plastic teeth were sandblasted with 110 µm aluminium oxide Korox at 2 bars (Bego, Bremer Goldschlägerein Wilh. Herbst GmbH & Co KG, Bremen, Germany; device: Renfert Classic Plus 3, Renfert GmbH, Hilzingen, Germany). A labelled diagram explaining the dental anatomy of the maxilla can be found online in Supplementary Figure S2. This process led to the adequate adhesion of the black two-component varnish applied to the plastic teeth (DuPont Refinish, Willich, Germany). After drying for 24 h, a second coat was applied. The black varnish provided an ideal contrast to white artificial plaque. Then, brackets (Mini 2000, Straight-Wire, Ormco, Orange, California, USA) were attached to the teeth by superglue (Henry Schein, Instant Fix, Melville, USA), and a wire (Niti-Wire round 0.012 inch, Orthana, Recklinghausen, Germany) was ligated with orthodontic ligatures (Dentalastics Personal, Dentauraum, Ispringen, Germany).

To ensure standardized and repositionable fixation of the teeth for photography, the teeth were placed into a plaster block after each cleaning stage. A total of three blocks were produced for the buccal, mesial (approximal surface directed towards the midline of the face) and distal (approximal surface directed away from the midline of the face) areas (Dento-stone 220, Dentona AG, special superhard plaster type 4, Dortmund, Germany). To make the blocks, the root of each tooth needed to be covered with wax to prevent areas divergence (Modelling wax, Henry Schein, Melville, USA). The teeth were set into plaster 2 cm apart from each other. To ensure an even height and distance, the crown of the tooth was fixed in A-silicone (polysiloxane, Henry Schein, Melville, USA). Twenty millilitres of water was added to the plaster (100 g) and it was stirred with a vacuum mixer (Motova SLA, BEGO AG, Bremen, Germany). Then, the root of each blocked tooth was set into the plaster. To enable easy insertion and removal of the teeth from the block, cavities were milled into the plaster block. Then, the blocks were trimmed to their final shape (Renfert GmbH, Hilzingen, Germany).

To standardize the evaluation, it was important that the artificial plaque did not peel off, to ensure high contrast with the black tooth surface, and that its composition did not change during the trial. A suspension of titanium dioxide (13 g) and isopropyl alcohol (28 millilitres) (VWR International GmbH, Langenfeld, Germany) was used to simulate the plaque. With a magnetic stirrer (IKA-Werke GmbH & Co. KG, Staufen, Germany), the two ingredients were mixed for 30 min, turning them into a viscous compound. Standardized conditions were maintained by using mechanical stirring and constant ingredient mixing ratio. After a resting time of 24 h, the plaque was applied manually to the teeth by one brushstroke with a clean brush. By tapping the tooth on the edge of the table, the plaque spread uniformly over the tooth surface. It was allowed to dry for 4 h. For each cleaning trial, one layer was applied.

### Cleaning process

**Figure 1:**
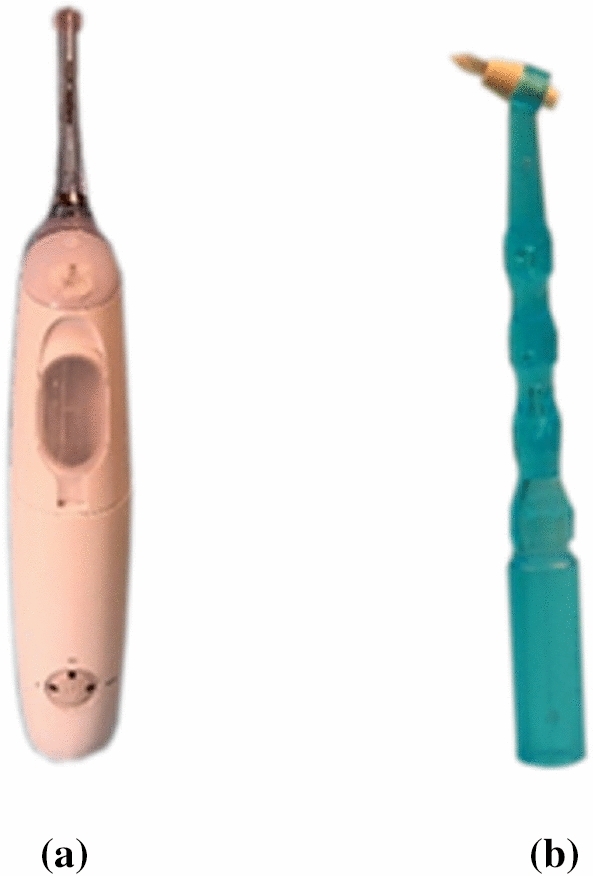
The Sonicare AirFloss Ultra HX8431 (**a**) and the sulcus brush I-Prox P (**b**) were used for cleaning.

Cleaning was accomplished by the Sonicare AirFloss Ultra HX8431 (Philips GmbH, Amsterdam, Netherlands) or by the sulcus brush I-Prox P (Miradent, Hager & Werken GmbH & Co. KG, Duisburg, Germany) (Fig. 1). Both devices were characterized by the fact that no proximal contact can be bypassed. The spray nozzle of the AirFloss device faces the buccal surface of the approximal area. By pressing a button, water sprays at a speed of 72 km per hour from the buccal to the oral surface facing the mouth cavity. Depending on the mode, one, two, or three spray bursts are applied. The sulcus brush consists of an approximately 14-cm-long handle with a tapered v-shaped brush. A new brush was used for each cleaning process. Cleaning was carried out by rotating movements in the buccal and approximal areas.

To standardize the cleaning processes, the AirFloss Ultra was always applied in contact with the tooth surface at a constant angle. The use of the devices was performed as recommended in the respective instruction manuals (see Supplementary Document S3). In contrast to a standard toothbrush, the I-Prox P brush is held at a right angle in relation to the buccal and approximal tooth surfaces. Because of the tilted head of the brush (120° angle), this cleaning position allows for gentle cleaning without irritating the gingiva. The brush was used with a contact pressure of two bars, and the application time was managed with a timer.

First, the buccal area was cleaned followed by the mesial and distal approximal surfaces. After each cleaning procedure, the brush of the I-Prox P was replaced to maintain a constant hardness of the cleaning filaments. The AirFloss pressure of the sprays was pre-set and did not change during the trial. Depending on the cleaning routine, the double burst mode was accomplished one (two spray bursts per area) and two times (four spray bursts per area). The nozzle tip of the AirFloss was placed horizontally between two teeth at the gum line while the activation button was pressed to deliver two and four bursts, respectively.

**Figure 2:**
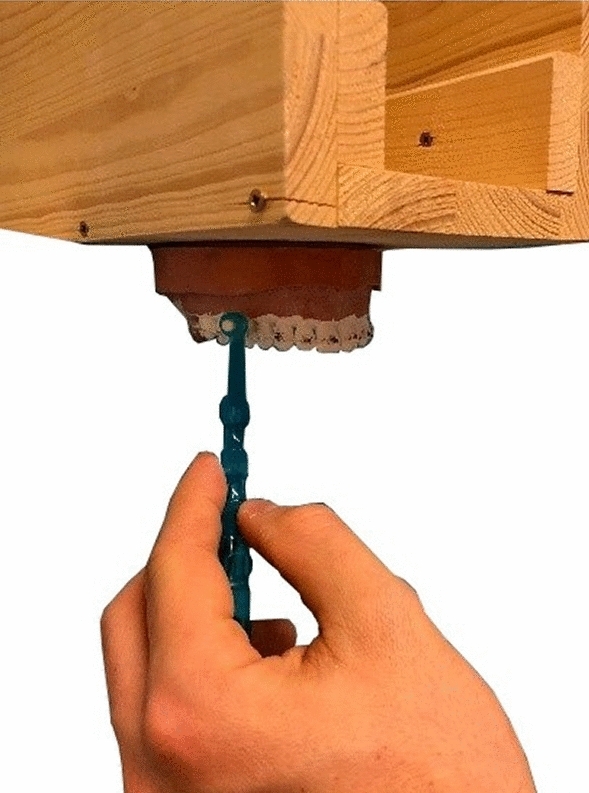
A specially built appliance was attached to the KaVo model to ensure standardized cleaning.

To achieve cleaning as close to reality as possible, the KaVo model was fixed to a special appliance. This setup allowed simulated cleaning of the upper teeth at eye level and from the front as if they were the teeth of the trial operator. Additionally, it prevented the model from moving during the process (Fig. 2).

Every cleaning stage was divided into four groups. Each group was repeated four times leading to a total of 16 cleaning procedures.

- The use of the I-Prox P brush for two seconds per area (I-2).
- The use of the I-Prox P brush for four seconds per area (I-4).
- The use of the AirFloss Ultra with two spray bursts per area (A-2).
- The use of the AirFloss Ultra with four spray bursts per area (A-4).

Each cleaning routine (I-2, I-4, A-2, A-4) included the cleaning of four incisors, two canines, four premolars, and four molars (see Supplementary Figure S2). Since only two canines were included in the KaVo model, an extra cleaning stage was carried out by cleaning only the two canine. Cleaning a total of 16 teeth in each cleaning routine and repeating each group four times led to an equal number of 64 evaluated teeth of each tooth type (incisors, canines, premolars, molars). All the cleaned teeth in all four groups totalled 256 teeth.

### Photograph editing

After each completed cleaning procedure, the teeth were removed from the KaVo model and placed into the plaster blocks. In a photobooth, photographs were taken by a Canon EOS 60D with an EF-S 18–25 mm IS II lens (Canon GmbH, Krefeld, Germany) at 62.2 cm.

Before applying titanium oxide to the teeth, a total of 12 masks of the buccal and approximal surfaces were produced, defining the areas considered for evaluation (Adobe Photoshop CS5, Adobe Systems Software Ireland Limited, Dublin, Ireland).

**Figure 3:**
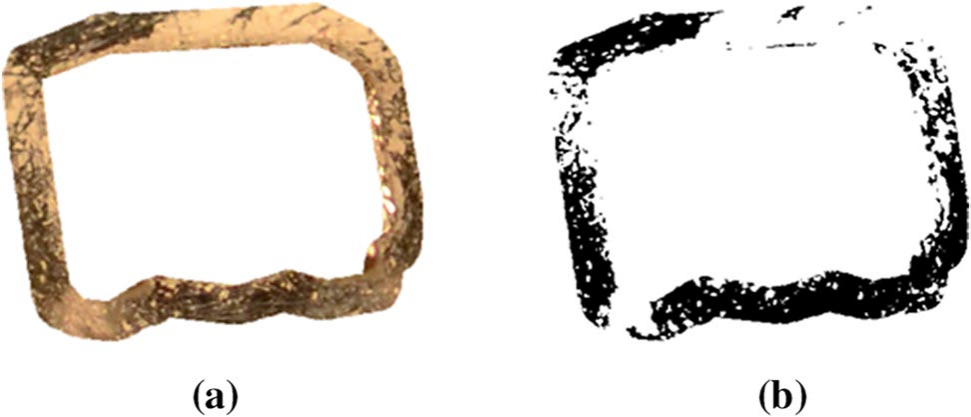
(**a**) shows the extracted photograph. (**b**) presents the black-and-white picture of the cleaned, white areas and the uncleaned, black areas.

After putting the teeth in the plaster blocks, the masks produced in advance were layered on the cleaned photographed areas. The extraction was conducted using Adobe Photoshop CS5. The generated cut-outs were evaluated with the ImageJ software program (National Institutes of Health, Bethesda, USA). By turning them into black-and-white pictures, the cleaned areas (black) were distinguishable from the uncleaned surfaces (white). The ratio of white pixels to the total number of pixels of the mask corresponded to the cleaning efficacy (Fig. 3).

### Outcome measures

The working hypothesis assumed that the AirFloss Ultra would achieve a higher cleaning efficacy than the sulcus brush I-Prox P.

### Statistical analysis

To produce significant results, the sample size was estimated with the G* Power 3.1.9.2 program^30^. A sample size of 32 teeth (16 teeth per group) was analysed with a statistical power of 0.8, an alpha error of 0.05, and an effect size of 1.04.

All data were analysed with the SPSS 25.0 software program (IBM Germany GmbH, Ehningen, Germany). All 256 teeth served as the bases for the statistical analysis. Each area of each tooth (mesial, distal, and buccal) was evaluated, leading to a total of 768 single values. For the descriptive statistics, the minimum, maximum, median, mean, and standard deviation were used. Kolmogorov–Smirnov test identified a normal distribution with an asymptomatic significance value of p = 0.2 for the mesial, distal, and buccal areas in all the cleaning groups. Within groups I-2, A-2, and A-4, a normal distribution with an asymptomatic significance value of p = 0.2 was found. The values of cleaning groups I-4 showed an asymptomatic significance value of p = 0.01. Subsequent ANOVAs and post hoc tests with Bonferroni correction were used to determine the significance between the cleaning groups at a significance level of p = 0.05.

## Results

The cleaning efficacy was based on the evaluation of 256 teeth. The mesial, distal, and buccal surface of every tooth was observed. Each cleaning procedure provided 14 buccal, 14 distal and 14 mesial photos. A total of 768 single pictures were obtained by conducting the two cleaning stages. The I-Prox brush achieved an overall cleaning efficacy of 26.87% (SD: 11.37) with a two-second application (I-2) and 43.73% (SD: 10.74) with a four-second application (I-4). The AirFloss managed to clean 34.93% (SD: 9.54) of the evaluated tooth surfaces with two sprays (A-2) and 56.78% (SD: 10.12) with four sprays (A-4). The results of the cleaning efficacy of the Sonicare AirFloss Ultra and the I-Prox P brush are described in Table 1.

**Table 1:** The means (in percent) and standard deviation (SD) of the cleaned areas of each cleaning group (I-Prox P brush for 2 s (I-2) and four seconds (I-4) and the Airflow for 2 sprays (A-2) and four sprays (A-4)).

Group	Total	Area of tooth			Type of tooth			
		Buccal	Mesial	Distal	Incisor	Canine	Premolar	Molar
I-2 (*n* = 14)	26.87 (11.37)	39.48 (5.45)	17.51 (5.73)	23.62 (8.27)	29.74 (10.51)	29.09 (7.08)	23.86 (14.07)	25.89 (11.33)
I-4 (*n* = 14)	43.73 (10.74)	53.84 (5.67)	37.01 (9.07)	40.35 (8.84)	48.51 (5.61)	45.29 (8.57)	38.01 (12.38)	43.89 (12.22)
A-2 (*n* = 14)	34.93 (9.54)	31.98 (6.34)	37.24 (12.43)	35.57 (8.76)	30.80 (6.34)	29.41 (8.92)	35.69 (7.99)	41.05 (11.05)
A-4 (*n* = 14)	56.78 (10.12)	53.18 (8.93)	59.71 (10.88)	57.44 (10.07)	49.47 (6.20)	56.50 (11.20)	60.87 (7.86)	60.13 (11.66)

**Figure 4:**
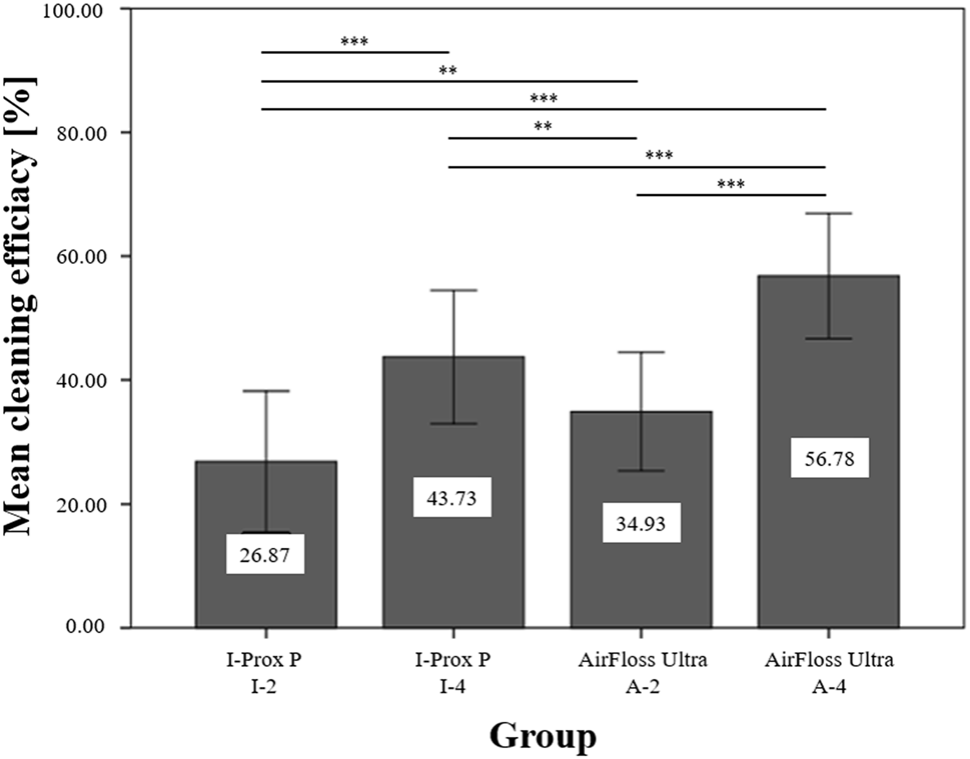
The mean total cleaning efficacy (in percent) with the standard deviation of the I-Prox P brush for two seconds (I-2) and four seconds (I-4) and the AirFloss Ultra with 2 sprays (A-2) and 4 sprays (A-4). Horizontal lines with “***” show significant differences between the devices with p < 0.001, and “**” indicates a significant difference with p < 0.01.

With a mean cleaned area of 56.78% (SD: 10.12%), the overall cleaning efficacy of the AirFloss Ultra (A-4) was significantly higher. The least efficient result was achieved by the I-Prox P brush (I-2), with a mean cleaned area of 26.87% (SD: 11.37%). The application of the AirFloss Ultra with two sprays (A-2) cleaned 34.93% (SD: 9.54%) of the tooth surface and the I-Prox P brush (I-4) for 4 s cleaned 43.73% of the tooth surface (SD: 10.74%). Significant differences existed between all groups, with p < 0.001 and p < 0.01 (Fig. 4).

**Figure 5:**
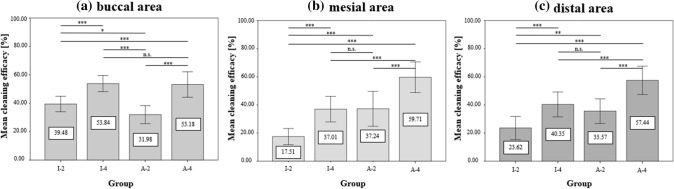
The means and standard deviation of the cleaning efficacy of the buccal (**a**), mesial (**b**) and distal (**c**) areas. Horizontal lines with “***” describe significant differences with p < 0.001, “**” with p < 0.01, and “*” with p < 0.05 between the I-Prox P brush applied for two (I-2) and four seconds (I-4) and the AirFloss with two (A-2) and four sprays (A-4). “n.s.” indicates no significant differences.

Significant differences between cleaning groups were not observed based on the mean cleaning efficacy of the cleaned area (buccal, mesial, distal) (Fig. 5). The corresponding mean and standard deviation of each application in relation to the tooth area are presented in Table 1.

**Figure 6:**
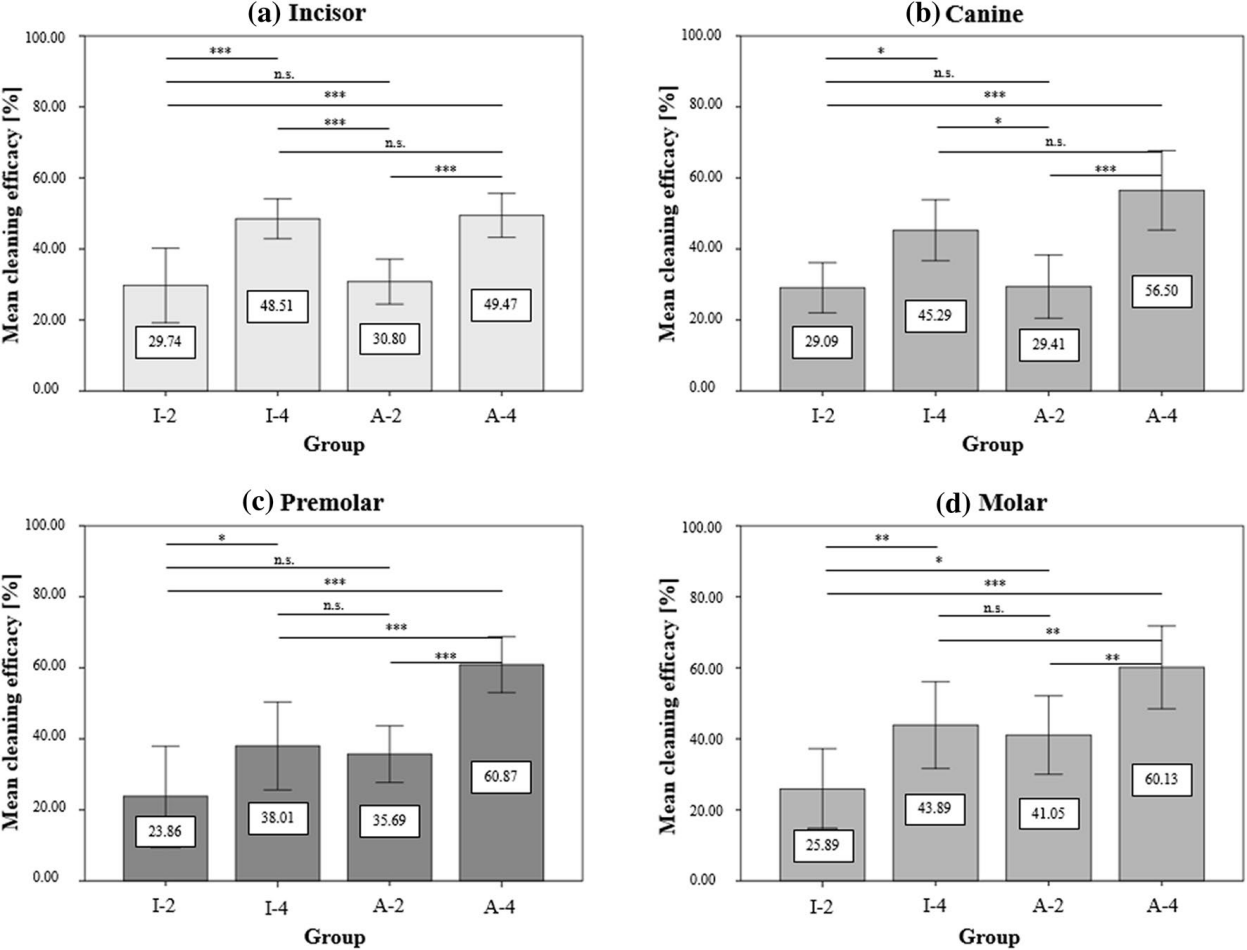
The means and standard deviation of the cleaning efficacy of the incisors (**a**), canines (**b**), premolars (**c**), and molars (**d**). Horizontal lines with “***” describe significant differences with p < 0.001, “**” with p < 0.01, and “*” with p < 0.05 between the I-Prox brush applied for two (I-2) and four seconds (I-4) as well as the AirFloss with two (A-2) and four sprays (A-4). “n.s.” indicates no significant differences.

Further significant differences in the mean cleaning efficacy between the applied devices can be seen with respect to the type of tooth (incisor, canine, premolar, and molar) (Fig. 6). The corresponding mean and standard deviation of each application with respect to tooth type are presented in Table 2.

**Table 2:** Maximum and minimum cleaning efficacy of the I-Prox P brush and the AirFloss Ultra cleaning devices at different application frequencies (two seconds (I-2)/sprays (A-2) vs. four seconds (I-4)/sprays (A-4)).

	Total	Area of tooth			Type of tooth			
		Buccal	Mesial	Distal	Incisor	Canine	Premolar	Molar
Maximum	A-4	A-4/I-4	A-4	A-4	A-4/I-4	A-4/I-4	A-4	A-4
Minimum	I-2	A-2	I-2	I-2	A-2/I-2	A-2/I-2	A-2/I-2	I-2

Table 2 presents an overview of the maximum and minimum cleaning efficacy of each tooth surface and tooth type achieved by the use of the I-Prox P brush for two (I-2) or four (I-4) seconds or the application of the AirFloss Ultra with two (A-2) or four (A-4) sprays.

## Discussion

The long duration of orthodontic therapy with fixed appliances usually causes patients to lose compliance with adequate oral hygiene^9,31^. This in vitro study compared the sulcus brush I-Prox P by Miradent to the Philips AirFloss Ultra device, which shows potential for easy and effective cleaning.

This study shows the advantages and disadvantages regarding their cleaning efficacy. However, uncertainty parameters associated with in vivo models, such as the dependency of compliance, cleaning routine, manual skills, and saliva composition, were eliminated. On the other hand, the plaque substitute and the teeth themselves do not correspond exactly to real conditions. To measure the cleaning efficacy more precisely, in vivo studies are required using measurable parameters such as the approximal plaque or the stage of inflammation of the interdental papilla^32^. Because of the greater plaque formation on the buccal sites of maxillary teeth compared to mandibular teeth, maxillary teeth are thought to be more difficult to clean than lower teeth^33^. For this reason, the maxillary model was chosen for the cleaning procedures.

During the cleaning process, the approximal areas are not visible to the person conducting the study. This study used plaster blocks to create standardized and reproducible conditions for the trial process. In addition, this setup enabled a precise digital analysis of the buccal and approximal areas. The evaluation and analysis of the percent of cleaning efficacy were performed by ImageJ software. Previous studies used this program to evaluate photographs, describing it as a reliable measurement tool^34,35^.

The simulation of dental plaque is one of the challenges of clinical studies. To standardize laboratory conditions, a well-established test method for the cleaning effectiveness of different devices was used^36^. Schätzle et al. also used titanium oxide to simulate dental biofilm^37^. Titanium oxide does not peel off during the cleaning procedure and shows high contrast to black-coloured teeth^38^. This method enables a precise evaluation of the cleaned areas. Although the plaque substitute does not show any other similarities to natural plaque, significant findings can be achieved^36,37,39^. However, in vivo studies are required to verify the results.

Previous studies have proven that cleaning devices remove plaque reliably in areas with direct contact between the device and the tooth surface^40^. This situation leads to the assumption that the cleaning result is dependent on the dimensions of the contact area of the cleaning device and the tooth surface. Within this study, the aim was not only to measure the efficacy of cleaning but also to illustrate all areas reached by the application of the tested devices.

### Statement 1: The overall cleaning performance of the AirFloss ultra is superior to that of the I-Prox P brush

The overall cleaning performance is a summary of the cleaning effects on the individual tooth surfaces (buccal, mesial, and distal) and tooth types (incisors, canines, premolars, and molars). The I-Prox P brush with a four-second application (I-4) achieved a comparable cleaning efficiency on the buccal surfaces and on the incisor and canine teeth compared to the AirFloss Ultra with four applications (A-4), but it performed significantly worse in the proximal and posterior areas. Since the overall cleaning effect is based on the summation of all tooth surfaces and tooth types, the overall cleaning efficacy of the AirFloss Ultra is significantly higher when used four times. The reasons for the significantly better cleaning effect of the AirFloss Ultra compared to the I-Prox P brush are mainly due to the structures and types of the cleaning devices. Key statements two and three provide a detailed statement about the results achieved.

### Statement 2: I-Prox P cleans more efficiently on buccal surfaces than on proximal surfaces

No significant difference was found regarding the cleaning efficacy of the I-Prox P brush (I-4) and AirFloss Ultra (A-4) when applied for four seconds and four sprays on the buccal surface, respectively. The slightly better cleaning result of the I-Prox P sulcus brush on the buccal surface can be attributed to its bristle design. Due to the conically tapered V-shaped bristle tufts, the brush tips also clean difficult-to-reach surfaces underneath the bracket-arch area. This reach results in a larger contact area between the tooth and bristle tips, which is crucial for efficient plaque removal^40^. In contrast, the wall shear stress of the AirFloss Ultra spray jet is not powerful enough at the margins to remove plaque adequately. In summary, the buccal area of the teeth can be cleaned as adequately by using the sulcus brush as by using the AirFloss Ultra.

However, the limitations of the I-Prox P brush appear when considering the cleaning of the proximal areas. In the present study, the proximal region was divided into mesial and distal areas. The evaluation showed a significant superiority of the AirFloss Ultra with four sprays (A-4) on both surfaces. The inferior result of the I-Prox P brush in the proximal areas is due to the special brush architecture of the sulcus brush. In contrast to its effect on buccal surfaces, the V-shaped design of the bristle tufts is unsuitable for cleaning the proximal surfaces because it does not conform to the shape of the interdental area. When the bristles are inserted, the incongruity between the bristle design and the interdental space causes them to lie closely on top of each other, preventing the cleaning of the orally located areas of the proximal space. This phenomenon is called the “umbrella effect”^40^. Additionally, the increase in insertion resistance leads to a drift of the brush tip towards the gingiva, which may cause injuries.

### Statement 3: the AirFloss ultra cleans premolars and molars more efficiently than incisors and canines

Table 2 illustrates that the AirFloss Ultra is significantly superior to the I-Prox P in cleaning premolars and molars. However, when cleaning incisors and canines, there was no significant difference between the two cleaning devices when applied four times and four seconds. The AirFloss Ultra cleans slightly less efficiently on the incisors and canines than in the premolar and molar areas. This outcome is due to the different morphology of the proximal region of the teeth. Compared to premolars and molars, the proximal region of the anterior and canine teeth is characterized by a larger apical and incisal area and mesial and distal extension. In contrast, the posterior teeth have greater extension in the vestibular and oral directions.

When cleaning with the AirFloss Ultra, only the areas reached by the spray are cleaned. The radius of the spray jet is determined by the spray head. Because of the greater expansion in the apical and incisal, mesial, and distal directions of the proximal surfaces of the anterior and canine teeth, areas that are not directly in the radius of the spray remain uncleaned. Since the proximal surfaces in the premolar and molar regions tend to have the greatest extension in the vestibular and oral directions, the cleaning power increases. The AirFloss Ultra is therefore most efficient in the premolar region.

A previous study used a calculation model to determine the force per area generated by a spray from the AirFloss and called it wall shear stress^41^. A spray of 1.7 kPa (kilopascal) was adequate to dissolve the adhesion of most of the bacterial colonies. Because of the tooth morphology, the wall shear stress decreases from the buccal to oral direction. The fluid flow generated by the AirFloss moves straight, without following the tooth anatomy. A wall shear stress of 2.7 kPa was determined at the vestibular site of the proximal region, which decreased to 1.7 kPa in the middle area and measured 0.3 kPa in the oral area. The minor cleaning effect of the AirFloss in the oral region can be confirmed by this study. The small spray radius of the AirFloss spray head justifies the minor cleaning efficacy in the anterior and canine regions compared to the posterior region. A larger spray head on the AirFloss Ultra might lead to superior cleaning performance in the anterior region. This hypothesis is confirmed by the results of the present study.

### Statement 4: repeated use of the cleaning device leads to increased cleaning efficacy

The lowest cleaning efficiency was achieved with a two-second (I-2) and two-spray (A-2) application of the cleaning device, contrary to the maximum cleaning efficiency achieved by four second (I-4) and four spray (A-4) repetitions. Thus, the percentage of the plaque removed can be improved by increasing the cleaning time or the number of sprays. The cleaning performance of the AirFloss Ultra is based on the wall shear stress developed by the spray. If this stress is greater than the adhesive strength of the plaque substitute, then the tooth surface is cleaned. Additionally, if the spray of the AirFloss device is applied several times, then the wall shear stress acts more frequently on the examined tooth surface. As a result, plaque particles that were not completely detached by the two sprays seemed to be removed by reapplying the wall shear stress. The reason for this outcome may be the reduction in the adhesive strength of the artificial plaque. A comparative study also assessed higher plaque removal when increasing the brushing time^42^. The cleaning performance of the I-Prox P brush behaves in a similar way. This confirms the generally accepted statement in the literature that cleaning is improved by extending the cleaning time^43,44^.

## Conclusion

Within the limitations of an in vitro study with plaque substitutes, the results of this study have shown that AirFloss Ultra and I-Prox P brushes achieve a reduction in plaque substitution in all cleaning cycles. They enable a cleaning process without bypassing the approximal contact point of the teeth. This is an important aspect for patients wearing fixed orthodontic appliances and helps simplify their daily oral hygiene. The significantly higher overall cleaning performance of the AirFloss Ultra compared to the I-Prox P brush found in this study provides an indication for recommending the AirFloss Ultra for oral hygiene during multibracket therapy. The extent to which the I-Prox P brush is suitable for plaque reduction in multibracket patients cannot be conclusively determined by the present study. Although the test method proved to be practicable and effective, additional studies must follow to clinically verify the results; specifically the cleaning efficiency of the I-Prox P brush in patients undergoing orthodontic treatment needs to be evaluated and the issue of compliance needs to be addressed. Our results indicated effective cleaning with different devices but the result may be very different than those of true teeth cleaning. Additionally, the cleaning performance was improved by a longer cleaning process or multiple applications. Considering this finding, when applying the tested devices, patients should be instructed to use the respective devices for a longer period or several times during each cleaning procedure.

## Supplementary Information

Supplementary Information 1.

Supplementary Information 2.

Supplementary Information 3.

## Data Availability

The authors confirm that the data supporting the findings of this study are available within the article and its supporting materials.
