# Supplementary material for: Comparative in vitro study of the cleaning efficacy of AirFloss ultra and I-Prox Sulcus brushes in an orthodontic phantom model
Source: Sci Rep. 2021 Jan 21;11:1921. doi: 10.1038/s41598-021-81603-y (PMC7820349; doi:10.1038/s41598-021-81603-y)
Supplement: Supplementary file 1 — Supplementary Information 1. [file 41598_2021_81603_MOESM1_ESM.pdf]

Comparative In Vitro Study of the Cleaning Efficacy of AirFloss Ultra and I-Prox Sulcus Brushes in an Orthodontic Phantom Model

Hanna Boes[1*], Sören Brüstle[1], Gholamreza Danesh[2], Stefan Zimmer[1], Mozhgan Bizhang[1]

[1] Department of Operative and Preventive Dentistry, Faculty of Health, Witten/Herdecke University, Witten, Germany

[2] Department of Orthodontics, Faculty of Health, Witten/Herdecke University, Witten, Germany

*** Corresponding author:**

Universität Witten/Herdecke

Alfred-Herrhausen-Str. 50

58455 Witten, Germany

Tel. +49 2302 926 626

Fax. +49 2302 926 681

E-Mail: Hanna.Boes@uni-wh.de

Dataset of the in-vitro study

Zahn	Gruppe	Zahnlokalisation	Zahnart	Zahnart_Gr	V_1
Z11_I2_b	1	1	1	112	17.819
Z12_I2_b	1	1	1	112	11.887
Z11_I2_m	1	2	1	112	16.189
Z12_I2_m	1	2	1	112	11.508
Z11_I2_d	1	3	1	112	17.910
Z12_I2_d	1	3	1	112	18.211
Z11_I4_b	2	1	1	114	22.088
Z12_I4_b	2	1	1	114	17.875
Z11_I4_m	2	2	1	114	37.449
Z12_I4_m	2	2	1	114	20.427
Z11_I4_d	2	3	1	114	49.062
Z12_I4_d	2	3	1	114	25.154
Z11_Ps2_b	3	1	1	122	8.912
Z12_Ps2_b	3	1	1	122	11.332
Z11_Ps2_m	3	2	1	122	17.042
Z12_Ps2_m	3	2	1	122	5.512
Z11_Ps2_d	3	3	1	122	50.787
Z12_Ps2_d	3	3	1	122	17.996
Z11_Ps4_b	4	1	1	124	18.698
Z12_Ps4_b	4	1	1	124	15.659
Z11_Ps4_m	4	2	1	124	50.946
Z12_Ps4_m	4	2	1	124	30.539
Z11_Ps4_d	4	3	1	124	51.903
Z12_Ps4_d	4	3	1	124	46.466
Z13_I2_b	1	1	2	212	15.037
Z13_I2_m	1	2	2	212	17.800
Z13_I2_d	1	3	2	212	22.140
Z13_I4_b	2	1	2	214	24.645
Z13_I4_m	2	2	2	214	32.400
Z13_I4_d	2	3	2	214	56.929
Z13_Ps2_b	3	1	2	222	16.125
Z13_Ps2_m	3	2	2	222	17.309
Z13_Ps2_d	3	3	2	222	57.107
Z13_Ps4_b	4	1	2	224	27.347
Z13_Ps4_m	4	2	2	224	30.669
Z13_Ps4_d	4	3	2	224	79.460
Z14_I2_b	1	1	3	312	17.859
Z15_I2_b	1	1	3	312	14.322
Z14_I2_m	1	2	3	312	12.916
Z15_I2_m	1	2	3	312	9.750
Z14_I2_d	1	3	3	312	17.743
Z15_I2_d	1	3	3	312	21.588
Z14_I4_b	2	1	3	314	20.045
Z15_I4_b	2	1	3	314	18.038
Z14_I4_m	2	2	3	314	46.404
Z15_I4_m	2	2	3	314	21.870
Z14_I4_d	2	3	3	314	20.537
Z15_I4_d	2	3	3	314	24.176
Z14_Ps2_b	3	1	3	322	11.332

Z15_Ps2_b	3	1	3	322	13.157
Z14_Ps2_m	3	2	3	322	73.375
Z15_Ps2_m	3	2	3	322	55.415
Z14_Ps2_d	3	3	3	322	51.131
Z15_Ps2_d	3	3	3	322	36.477
Z14_Ps4_b	4	1	3	324	21.053
Z15_Ps4_b	4	1	3	324	16.995
Z14_Ps4_m	4	2	3	324	95.121
Z15_Ps4_m	4	2	3	324	91.012
Z14_Ps4_d	4	3	3	324	70.132
Z15_Ps4_d	4	3	3	324	66.686
Z16_I2_b	1	1	4	412	19.885
Z17_I2_b	1	1	4	412	14.851
Z16_I2_m	1	2	4	412	19.571
Z17_I2_m	1	2	4	412	17.944
Z16_I2_d	1	3	4	412	9.750
Z17_I2_d	1	3	4	412	36.533
Z16_I4_b	2	1	4	414	27.135
Z17_I4_b	2	1	4	414	20.896
Z16_I4_m	2	2	4	414	38.716
Z17_I4_m	2	2	4	414	41.390
Z16_I4_d	2	3	4	414	41.130
Z17_I4_d	2	3	4	414	61.719
Z16_Ps2_b	3	1	4	422	16.396
Z17_Ps2_b	3	1	4	422	18.553
Z16_Ps2_m	3	2	4	422	65.878
Z17_Ps2_m	3	2	4	422	64.950
Z16_Ps2_d	3	3	4	422	53.810
Z17_Ps2_d	3	3	4	422	27.650
Z16_Ps4_b	4	1	4	424	18.630
Z17_Ps4_b	4	1	4	424	24.969
Z16_Ps4_m	4	2	4	424	102.798
Z17_Ps4_m	4	2	4	424	88.535
Z16_Ps4_d	4	3	4	424	62.043
Z17_Ps4_d	4	3	4	424	62.660
Z21_I2_b	1	1	1	112	15.265
Z22_I2_b	1	1	1	112	15.176
Z21_I2_m	1	2	1	112	19.691
Z22_I2_m	1	2	1	112	27.917
Z21_I2_d	1	3	1	112	30.744
Z22_I2_d	1	3	1	112	23.925
Z21_I4_b	2	1	1	114	24.227
Z22_I4_b	2	1	1	114	17.996
Z21_I4_m	2	2	1	114	42.085
Z22_I4_m	2	2	1	114	29.097
Z21_I4_d	2	3	1	114	54.586
Z22_I4_d	2	3	1	114	27.385
Z21_Ps2_b	3	1	1	122	13.612
Z22_Ps2_b	3	1	1	122	9.241
Z21_Ps2_m	3	2	1	122	26.857

Z22_Ps2_m	3	2	1	122	28.813
Z21_Ps2_d	3	3	1	122	36.153
Z22_Ps2_d	3	3	1	122	24.840
Z21_Ps4_b	4	1	1	124	16.853
Z22_Ps4_b	4	1	1	124	13.905
Z21_Ps4_m	4	2	1	124	46.836
Z22_Ps4_m	4	2	1	124	32.814
Z21_Ps4_d	4	3	1	124	69.171
Z22_Ps4_d	4	3	1	124	32.342
Z23_I2_b	1	1	2	212	12.713
Z23_I2_m	1	2	2	212	18.799
Z23_I2_d	1	3	2	212	36.481
Z23_I4_b	2	1	2	214	23.833
Z23_I4_m	2	2	2	214	41.023
Z23_I4_d	2	3	2	214	52.749
Z23_Ps2_b	3	1	2	222	10.266
Z23_Ps2_m	3	2	2	222	28.635
Z23_Ps2_d	3	3	2	222	45.153
Z23_Ps4_b	4	1	2	224	30.994
Z23_Ps4_m	4	2	2	224	57.074
Z23_Ps4_d	4	3	2	224	60.788
Z24_I2_b	1	1	3	312	16.747
Z25_I2_b	1	1	3	312	13.250
Z24_I2_m	1	2	3	312	33.421
Z25_I2_m	1	2	3	312	11.547
Z24_I2_d	1	3	3	312	17.047
Z25_I2_d	1	3	3	312	41.615
Z24_I4_b	2	1	3	314	16.439
Z25_I4_b	2	1	3	314	16.327
Z24_I4_m	2	2	3	314	55.051
Z25_I4_m	2	2	3	314	35.768
Z24_I4_d	2	3	3	314	33.911
Z25_I4_d	2	3	3	314	47.082
Z24_Ps2_b	3	1	3	322	15.299
Z25_Ps2_b	3	1	3	322	9.241
Z24_Ps2_m	3	2	3	322	67.278
Z25_Ps2_m	3	2	3	322	38.397
Z24_Ps2_d	3	3	3	322	33.650
Z25_Ps2_d	3	3	3	322	45.271
Z24_Ps4_b	4	1	3	324	17.697
Z25_Ps4_b	4	1	3	324	18.771
Z24_Ps4_m	4	2	3	324	99.195
Z25_Ps4_m	4	2	3	324	54.573
Z24_Ps4_d	4	3	3	324	82.665
Z25_Ps4_d	4	3	3	324	72.201
Z26_I2_b	1	1	4	412	24.078
Z27_I2_b	1	1	4	412	16.745
Z26_I2_m	1	2	4	412	23.595
Z27_I2_m	1	2	4	412	25.402
Z26_I2_d	1	3	4	412	35.439

Z27_I2_d	1	3	4	412	40.695
Z26_I4_b	2	1	4	414	27.246
Z27_I4_b	2	1	4	414	17.922
Z26_I4_m	2	2	4	414	43.321
Z27_I4_m	2	2	4	414	31.946
Z26_I4_d	2	3	4	414	56.616
Z27_I4_d	2	3	4	414	68.514
Z26_Ps2_b	3	1	4	422	14.961
Z27_Ps2_b	3	1	4	422	9.477
Z26_Ps2_m	3	2	4	422	45.273
Z27_Ps2_m	3	2	4	422	66.635
Z26_Ps2_d	3	3	4	422	66.608
Z27_Ps2_d	3	3	4	422	47.408
Z26_Ps4_b	4	1	4	424	31.063
Z27_Ps4_b	4	1	4	424	28.491
Z26_Ps4_m	4	2	4	424	73.753
Z27_Ps4_m	4	2	4	424	92.121
Z26_Ps4_d	4	3	4	424	91.426
Z27_Ps4_d	4	3	4	424	55.732

V_2	V_3	V_4	V_5	V_6	V_7	V_8
12.508	19.398	20.582				
14.197	14.238	13.658				
28.695	20.903	19.736				
19.789	9.285	14.011				
25.088	21.815	28.505				
22.137	16.662	23.916				
24.868	22.573	28.134				
20.279	15.002	15.627				
51.402	50.584	48.571				
44.557	36.453	32.875				
36.650	75.976	63.694				
29.669	42.164	34.983				
16.870	16.679	18.215				
8.494	7.756	11.609				
32.014	24.472	35.871				
15.228	27.828	13.366				
55.803	49.443	52.009				
35.960	29.588	27.716				
21.155	20.923	24.810				
20.038	22.419	19.222				
58.757	54.739	38.749				
30.172	31.170	49.914				
57.203	59.534	60.482				
55.101	36.947	45.950				
10.012	18.148	21.216	21.641	21.238	18.361	23.997
18.148	10.370	25.594	23.437	26.020	22.503	17.635
21.720	24.933	33.763	25.357	37.871	36.708	29.085
23.096	29.783	26.453	25.974	30.684	30.440	31.758
36.377	34.610	30.226	33.393	31.353	31.179	32.785
46.854	56.207	52.744	48.453	52.674	41.094	42.618
19.225	11.235	14.881	20.834	20.929	19.231	20.760
17.700	15.065	12.484	28.253	18.222	22.924	18.757
42.302	58.451	49.591	61.589	62.152	67.584	63.519
38.752	35.942	27.906	39.010	41.615	41.611	45.996
43.628	30.282	40.752	42.841	38.260	46.846	49.757
85.156	86.664	83.232	67.206	69.779	74.778	80.381
16.436	16.842	19.693				
13.086	16.204	13.608				
21.173	24.143	30.005				
7.731	13.322	10.560				
13.300	12.913	17.121				
14.986	18.334	18.843				
18.321	20.722	19.893				
21.256	19.699	16.304				
47.410	45.206	51.345				
19.413	26.868	20.638				
28.399	31.894	30.409				
30.579	38.299	37.611				
11.790	10.879	7.241				

9.784	12.216	8.804
60.550	68.005	59.858
39.231	32.924	56.112
66.890	56.041	39.535
48.038	19.152	24.566
20.963	15.351	13.767
17.635	25.601	24.083
95.922	93.729	80.459
80.617	70.019	81.385
86.645	78.353	83.805
87.884	88.683	76.823
26.171	21.534	26.818
7.709	12.768	14.337
27.498	22.012	27.570
12.461	7.447	22.727
9.555	14.320	18.628
24.479	38.018	37.280
27.791	33.835	38.102
21.198	22.944	20.634
46.331	47.764	32.465
33.316	37.771	34.417
33.904	53.599	56.904
50.582	56.552	62.595
8.887	11.587	15.260
23.337	15.643	17.527
62.028	66.579	85.243
83.415	75.793	71.811
19.207	51.917	44.849
47.867	36.582	41.553
23.084	33.779	25.366
23.383	27.802	28.526
101.796	81.624	82.389
96.879	101.580	87.969
91.267	77.214	85.688
70.794	52.043	68.447
16.808	19.445	19.676
17.046	17.914	11.975
12.662	13.990	12.065
11.206	23.454	18.863
44.791	52.219	33.374
25.330	19.988	23.168
26.711	20.847	20.652
18.612	19.739	20.220
26.274	54.834	56.323
31.590	45.412	46.134
56.347	56.452	60.706
37.791	35.916	30.955
11.939	12.123	11.570
9.439	11.123	4.198
37.845	23.832	33.083

32.088	31.534	5.403				
39.316	42.902	30.827				
22.567	19.398	29.436				
18.211	13.319	14.000				
11.757	17.802	16.333				
40.987	52.619	44.997				
51.252	45.614	36.199				
70.655	58.239	44.028				
45.672	49.720	34.785				
19.822	16.614	21.440	16.026	21.135	16.461	22.496
19.980	26.042	30.252	21.658	26.998	31.659	29.633
45.236	32.339	33.018	51.697	50.828	43.350	46.675
28.775	26.545	27.818	23.897	30.293	29.769	26.979
37.089	53.646	54.630	35.935	46.978	36.843	34.134
64.261	65.173	67.194	65.266	64.780	65.017	58.074
8.227	16.994	9.057	6.954	16.343	12.286	14.621
11.727	14.365	9.748	24.468	43.701	18.385	54.116
58.160	2.379	24.621	45.380	37.817	40.234	41.197
32.103	17.347	17.146	24.207	33.028	26.818	34.746
67.248	59.811	56.409	62.112	56.603	62.665	58.547
78.831	57.139	65.653	59.979	59.756	75.814	59.272
16.188	17.028	13.805				
14.816	13.726	10.863				
22.172	30.363	36.971				
10.114	7.328	5.310				
16.654	15.149	13.256				
42.410	30.944	35.224				
17.126	22.519	17.931				
17.789	19.682	21.105				
55.216	83.620	88.994				
26.994	31.919	45.402				
37.228	32.558	28.494				
66.915	53.439	60.560				
16.858	12.308	13.223				
17.564	15.879	12.516				
72.328	62.448	74.371				
34.719	46.467	10.222				
61.726	26.168	59.254				
25.587	4.011	57.222				
17.371	17.105	18.580				
20.687	23.579	22.303				
104.759	92.733	103.172				
72.723	68.699	64.854				
77.606	71.184	93.092				
74.920	81.503	94.803				
19.569	23.896	25.808				
11.931	16.267	10.324				
8.468	28.754	23.532				
27.427	23.310	26.166				
47.251	38.832	43.612				

42.529	40.756	40.627
33.839	35.615	46.518
23.791	25.824	16.508
30.862	57.712	52.661
40.382	44.815	44.367
60.251	58.854	67.448
71.864	81.231	60.099
21.142	19.949	20.711
23.052	10.675	16.409
64.170	54.299	68.425
65.643	62.747	81.046
72.423	46.913	81.651
50.282	47.403	45.103
28.248	28.697	26.744
26.332	25.718	26.656
76.159	92.528	66.513
110.166	97.833	109.315
88.000	84.444	91.224
60.013	51.882	63.709

gesamte Pixelzahl
40.126
36.594
101.752
76.811
120.597
75.600
40.126
36.594
101.752
76.811
120.597
75.600
40.126
36.594
101.752
76.811
120.597
75.600
40.126
36.594
101.752
76.811
120.597
75.600
50.564
97.543
131.427
50.564
97.543
131.427
50.564
97.543
131.427
50.564
97.543
131.427
37.206
37.217
146.931
112.101
118.461
128.321
37.206
37.217
146.931
112.101
118.461
128.321
37.206

37.217
146.931
112.101
118.461
128.321
37.206
37.217
146.931
112.101
118.461
128.321
53.502
43.543
123.218
140.353
121.547
144.696
53.502
43.543
123.218
140.353
121.547
144.696
53.502
43.543
123.218
140.353
121.547
144.696
53.502
43.543
123.218
140.353
121.547
144.696
40.490
36.533
102.133
76.714
120.001
75.862
40.490
36.533
102.133
76.714
120.001
75.862
40.490
36.533
102.133

76.714
120.001
75.862
40.490
36.533
102.133
76.714
120.001
75.862
50.689
97.129
131.662
50.689
97.129
131.662
50.689
97.129
131.662
50.689
97.129
131.662
37.355
37.366
146.742
112.176
118.269
128.628
37.355
37.366
146.742
112.176
118.269
128.628
37.355
37.366
146.742
112.176
118.269
128.628
37.355
37.366
146.742
112.176
118.269
128.628
53.132
43.369
123.616
140.939
121.511

144.972
53.132
43.369
123.616
140.939
121.511
144.972
53.132
43.369
123.616
140.939
121.511
144.972
53.132
43.369
123.616
140.939
121.511
144.972
