# Supplementary figures and images for: Comparative in vitro study of the cleaning efficacy of AirFloss ultra and I-Prox Sulcus brushes in an orthodontic phantom model
Source: Sci Rep. 2021 Jan 21;11:1921. doi: 10.1038/s41598-021-81603-y (PMC7820349; doi:10.1038/s41598-021-81603-y)

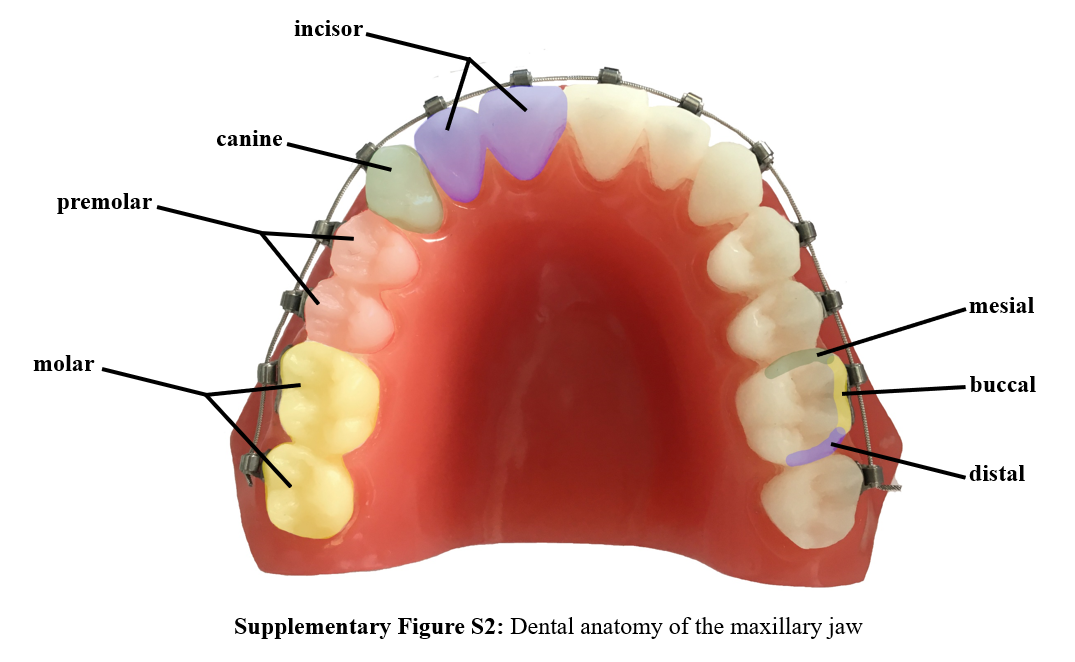

Supplement: Supplementary file 2 — Supplementary Information 2. [file 41598_2021_81603_MOESM2_ESM.tiff]
