# Supplementary material for: Comparative in vitro study of the cleaning efficacy of AirFloss ultra and I-Prox Sulcus brushes in an orthodontic phantom model
Source: Sci Rep. 2021 Jan 21;11:1921. doi: 10.1038/s41598-021-81603-y (PMC7820349; doi:10.1038/s41598-021-81603-y)
Supplement: Supplementary file 3 — Supplementary Information 3. [file 41598_2021_81603_MOESM3_ESM.pdf]

Comparative In Vitro Study of the Cleaning Efficacy of AirFloss Ultra and I-Prox Sulcus Brushes in an Orthodontic Phantom Model

Hanna Boes[1*], Sören Brüstle[1], Gholamreza Danesh[2], Stefan Zimmer[1], Mozhgan Bizhang[1]

[1] Department of Operative and Preventive Dentistry, Faculty of Health, Witten/Herdecke University, Witten, Germany

[2] Department of Orthodontics, Faculty of Health, Witten/Herdecke University, Witten, Germany

*** Corresponding author:**

Universität Witten/Herdecke

Alfred-Herrhausen-Str. 50

58455 Witten, Germany

Tel. +49 2302 926 626

Fax. +49 2302 926 681

E-Mail: Hanna.Boes@uni-wh.de

Instruction manuals of the tested devices

- ✓ **Thorough and gentle cleaning**
- ✓ **Storage compartment**
- ✓ **Set with 4 brushes**

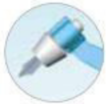

❶ V-shape
The pointed bristle cleans the periodontium thoroughly, effectively and gently

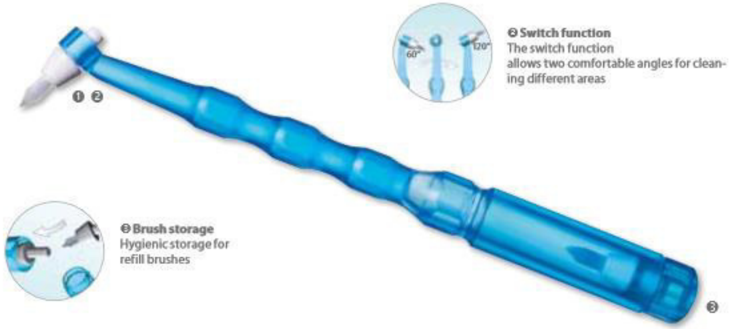

❷ Switch function
The switch function allows two comfortable angles for cleaning different areas

❸ Brush storage
Hygienic storage for refill brushes

I-Prox® P

Contains sulcus brush and four brushes

Neither toothbrushes, nor floss or interdental brushes clean every part of the mouth, such as:

- Sensitive exposed gingiva
- Gingival pockets
- Prosthetic works (bridges, crowns, implants, etc.)

The miradent sulcus brush with its finger-grip handle however, thoroughly and gently cleans areas that can neither be reached by toothbrush nor by interdental brush. The exchangable brushes are V-shaped and can be stored in the back of the holder.

Design	REF
I-Prox P, trans. blue	631 032
I-Prox P, trans. pink	631 033
I-Prox P Refill brushes, 4 pcs.	631 034

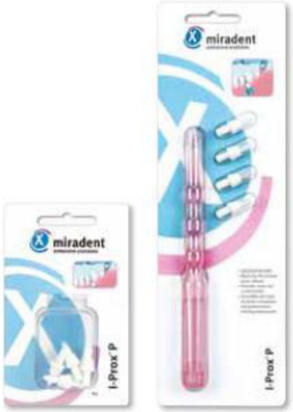

Applications:

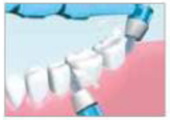
Molars

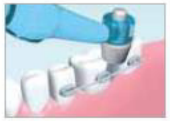
Brackets

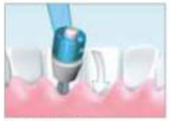
Gingival pockets

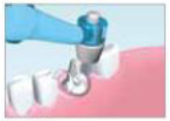
Implants

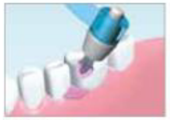
Applications

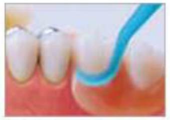
Removal of debris

- ✓ **Gentle and effective cleaning**
- ✓ **Removal of debris**
- ✓ **Easy home check-up**

I-Prox® Care Set

Interdental care set

For a complete cleaning of the oral cavity the combined use of the pointed I-Prox P brush and the double-ended toothscraper I-Prox C is advisable. This way, even hard to reach areas with debris can be cleaned gently. Following the cleaning process, the anti-fog coated mouth mirror makes it easy to inspect the mouth cavity.

All above-mentioned products are now available in a convenient interdental care set.

Design	REF
I-Prox Care Set, trans. blue	630 160

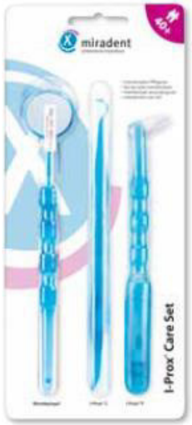

Always here to help you

Register your product and get support at
www.philips.com/welcome

Question?
Contact
Philips

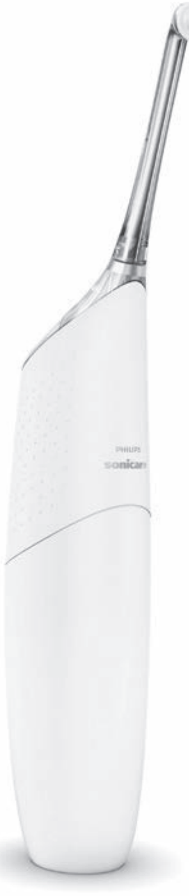

AirFloss Ultra

PHILIPS
sonicare

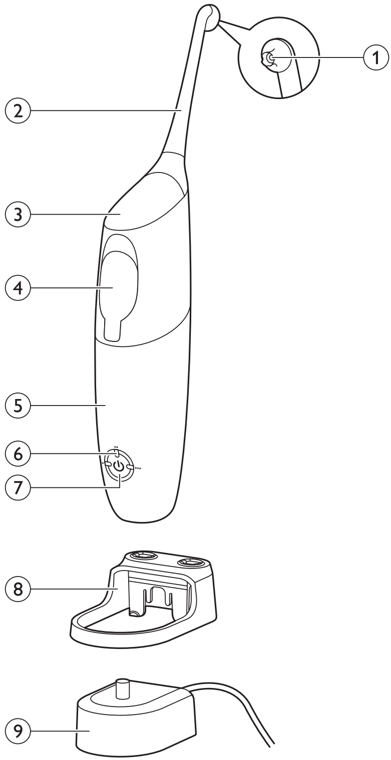
1
2
3
4
5
6
7
8
9

Philips Sonicare AirFloss Ultra

ENGLISH 4

Introduction

Congratulations on your purchase and welcome to Philips! To fully benefit from the support that Philips offers, register your product at **www.philips.com/welcome**.
Use AirFloss Ultra with your favorite mouthwash as part of your oral care routine. In addition to brushing, AirFloss Ultra helps reduce the plaque in between teeth in order to improve your gum health.

IMPORTANT SAFEGUARDS

READ ALL INSTRUCTIONS BEFORE USE

DANGERS

To reduce the risk of electrocution:

- Do not place or store product where it can fall or be pulled into water.
- Do not place or drop charger into water or other liquids, unplug the charger immediately if this should occur.
- After cleaning, make sure the charger is completely dry before you connect it to an electrical outlet.

WARNINGS

To reduce the risk of burns, electrocution, fire, or physical injury:

- Do not use isopropyl alcohol or other cleaning fluids in the reservoir as it may be an ingestion hazard.
- Do not use attachments other than those recommended by the manufacturer.
- Do not use hot water in the reservoir to avoid physical injury.
- Do not use the charger outdoors.
- Do not drop or insert any foreign objects into the reservoir.
- Use only charger type HX6100 provided with the AirFloss Ultra to recharge the battery.
- Never force the plug into an electrical outlet; if the plug does not easily fit into the electrical outlet, discontinue use.
- Keep the cord away from heated surfaces.
- Discontinue use if the nozzle, handle, or charger body/cord appears damaged in any way.
- The mains cord cannot be replaced. If the mains cord is damaged, discard the charger.
- This product contains no user-serviceable parts. Refer to chapter 'Warranty and support' if the product no longer works properly or needs repair.
- Use this product only for its intended use as described in this document or as recommended by your dental professional.
- This product is not intended for use by children and people with reduced physical, sensory, or mental capabilities, or lack of experience and knowledge, unless they have been given supervision or instruction concerning use of the product and understand the hazards involved.

- Children should be supervised to ensure that they do not play with the product.
- AirFloss Ultra is a personal care device and is not intended for use on multiple patients in a dental office or institution.
- Do not direct the spray under the tongue or into the ear, nose, eye, or other sensitive areas. This product may cause serious damage to these areas. See chapter 'Using your AirFloss Ultra' for correct usage.
- Avoid applying excessive pressure on the nozzle tip of the nozzle.

MEDICAL WARNINGS

- Consult your dentist before you use this product if you have had recent oral or gum surgery, suffer from an ongoing dental condition, had extensive dental work in the previous two (2) months, or have suspected issues with your dental work (filings, crowns, etc.).
- Contact your dental professional if excessive bleeding occurs after using this product or bleeding continues to occur after four (4) weeks of use.
- Discontinue use of this product and contact a physician/dentist if discomfort or pain is experienced.
- This product complies with the safety standards for electromagnetic devices. If you have a pacemaker or other implanted device, contact your physician or the device manufacturer prior to use.
- Consult your physician if you have other medical concerns.

Electromagnetic fields (EMF)
This Philips product complies with all applicable standards and regulations regarding exposure to electromagnetic fields.

SAVE THESE INSTRUCTIONS

Your Sonicare AirFloss Ultra (Fig. 1)

1 Nozzle tip
2 AirFloss Ultra nozzle
3 Activation button
4 Reservoir and reservoir cover
5 Handle
6 Charge & burst mode indicators
 - Single burst: 1 LED
 - Double burst: 2 LEDs
 - Triple burst: 3 LEDs
7 Power/mode button
8 Removable nozzle holder (select models)
9 Charger

Note: The contents of the box may vary based on the model purchased.

Charging

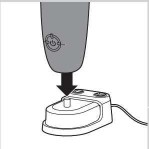

Charge AirFloss Ultra for 24 hours before first use.

1 Put the plug of the charger in the electrical outlet.

2 Place the handle on the charger.

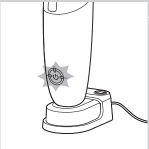

◗ Charging process:
- 1 green LED: 33% charged.
- 2 green LEDs: 66% charged.
- 3 green LEDS: 100% charged.

Note: When it is fully charged, AirFloss Ultra has an operating time of up to 11 days when it is used in triple burst mode, and up to 33 days when it is used in single burst mode.

Note: When the charge indicators flash yellow, the battery is low and needs to be recharged (fewer than 3 uses left).

Getting started

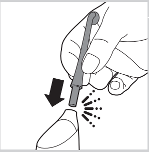

1 Align the nozzle and press the nozzle down onto the handle ('click').

Note: We advise you to place the tip facing the front of the handle as the illustration shows, but the nozzle can be placed in the handle either way without affecting the function. You can try both options and use what works best for you in your oral care routine.

Note: Replace the nozzle (HX8032, HX8033) every 6 months for optimal results. Also replace the nozzle if it becomes loose or no longer 'clicks' onto the handle.

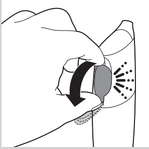

Note: The AirFloss Ultra only supports AirFloss Ultra nozzles. Do not attempt to use non-AirFloss Ultra nozzles on the AirFloss Ultra handle.

2 Open the cover of the reservoir ('click').

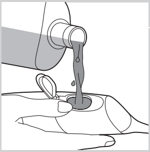

3 Fill the reservoir with mouthwash or water.

Tip: For the best result and a fresher experience, we advise you to use mouthwash with your AirFloss Ultra.

Note: Do not use mouthwash that contains isopropyl myristate (e.g. Dentyl Active) or high concentrations of essential oils, as it may damage the product. This includes all mouthwash sold in glass and ceramic packaging.

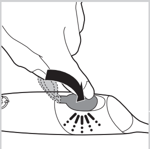

4 Close the cover of the reservoir by pressing ('click').

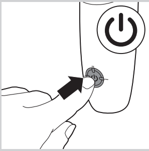

5 Press the power/mode button to turn on the product.

- ◗ The LEDs illuminate.

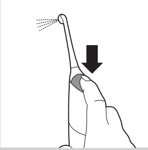

6 Press and hold the activation button until spray comes out of the tip of the nozzle.

Using your AirFloss Ultra

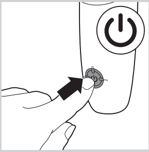

1 Make sure the AirFloss Ultra is turned on. If it is off, press and release the power/mode button to turn on the product.

- You can change the modes to have the desired amounts of bursts each time you press the activation button. For optimal results, use the triple burst mode (the default setting) from both the outside and inside of your teeth.
- To change modes, press the power/mode button to cycle through the modes until you reach your desired mode. The LEDs show the selected mode:

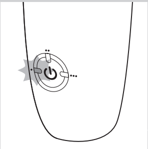

- Single burst: 1 LED

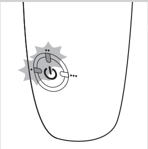

- Double burst: 2 LEDs

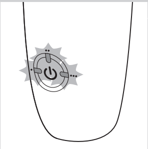

- Triple burst: 3 LEDs

Note: The AirFloss Ultra remembers what mode was selected last and it is ready for the next use. If your AirFloss Ultra becomes fully drained of power, it will go back to the triple burst default mode once it has been recharged.

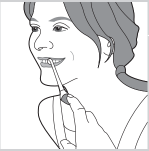

2 Place the nozzle tip between two teeth at your gum line. Slightly close your lips over the nozzle when it is in place, to avoid splashing.

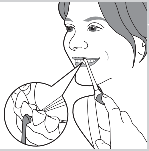

3 Press the activation button to deliver 1-3 bursts (depending on the selected mode) of air and micro-droplets of mouthwash or water between the teeth.

4 Slide the nozzle tip along the gum line until you feel it settle between the next two teeth.

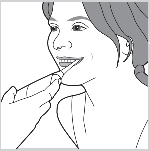

5 Continue this procedure for all spaces between your teeth, including behind your back teeth.

Note: Refill the reservoir as needed.

Auto-burst function

Hold the activation button to deliver bursts continuously, approximately one second between set of bursts.

1 To turn off AirFloss Ultra, press and hold the power/mode button for one second.

Automatic shut-off function

The AirFloss Ultra automatically turns off if it has not been used for one minute.

Cleaning

If the AirFloss Ultra becomes clogged, or to clean the inside of the AirFloss Ultra, fill the reservoir with warm water and press the activation button until the reservoir is empty.

Do not clean the nozzle, handle or charger in the dishwasher.

Do not use cleaning agents to clean the product. The formulation or strength of some agents may damage the product.

Handle and nozzle

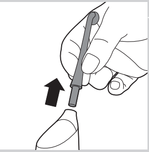

1 Remove the AirFloss Ultra nozzle from the handle. Rinse the nozzle after each use to eliminate the residue left in it.

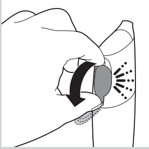

2 Open the reservoir and rinse it under a faucet to eliminate residue.

Tip: You can also use a cotton swab to wipe the reservoir and remove the remaining residue.

Note: Do not immerse the handle in water.

Note: Failure to remove residue from the nozzle or reservoir may result in an unhygienic condition.

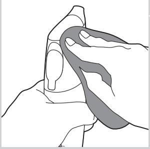

3 Wipe the entire surface of the handle with a damp cloth.

Charger

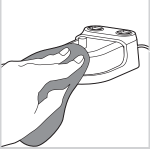

1 Unplug the charger before you clean it.

2 Wipe the surface of the charger with a damp cloth.

Storage

If you are not going to use AirFloss Ultra for an extended period of time, follow the steps below.

1 **Open the cover of the reservoir and empty it.**

2 **Press the activation button until no more spray comes out of the nozzle.**

3 **Unplug the charger.**

4 **Clean AirFloss Ultra nozzle, handle and charger. Refer to chapter 'Cleaning'.**

5 **Store AirFloss Ultra in a cool, dry place away from direct sunlight.**

Disposal

This product may contain lead and mercury. Disposal of these materials may be regulated due to environmental considerations. For disposal or recycling information, please contact your local authorities or visit **www.recycle.philips.com**.
This product contains batteries:

- Dispose of batteries properly. Do not incinerate. Batteries may explode if overheated.
- Do not wrap in metal or aluminum foil. Tape the waste battery terminals before discarding.
- It is suggested that you contact your local town or city to determine proper battery redemption site(s) in your area.
- Please visit **www.call2recycle.org** for additional information on a recycling center in your area for rechargeable batteries.

Removing the rechargeable battery

Please note that this process is not reversible.

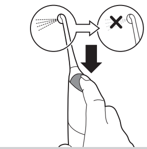

1 To deplete the battery, repeatedly press the activation button until the AirFloss Ultra no longer produces any bursts of air.

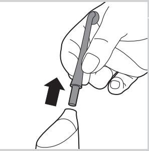

2 Remove the AirFloss Ultra nozzle from the handle.

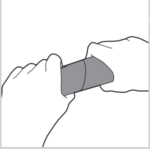

3 Firmly grab the handle with one hand at the top and one hand on the bottom.

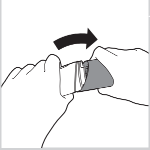

4 Snap the handle into two parts.

Note: This step requires a good amount of physical force. The top and bottom half of the handle should be separated once you snap the handle.

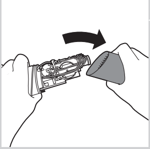

5 Remove the bottom half of the handle.

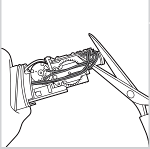

6 Cut all 6 wires on the internal component with scissors.

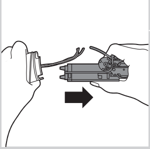

7 Completely pull apart the internal component from the top part of the handle.

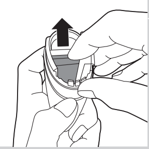

8 The battery is located inside the top half of the handle. Remove the battery and dispose of it properly (not in household waste).

Warranty and support

Philips warrants its products for two years after the date of purchase. Register your product at **www.philips.com/support**. Defects due to faulty materials and workmanship will be replaced at Philips expense provided that convincing proof of purchase in the qualifying period is provided. Use of unauthorized replacement parts will void this warranty. Contact our Consumer Care Center at 1-800-682-7664 (North America), outside North America contact your local Philips Consumer Care Center.
Internet information: **www.sonicare.com** (North America) or **www.philips.com/support** (outside North America).

WARRANTY EXCLUSIONS

What is not covered under warranty:
- AirFloss Ultra nozzle.
- Damage caused by misuse, abuse, neglect, alterations or unauthorized replacement.
- Normal wear and tear, including chips, scratches, abrasions, discoloration or fading.

IMPLIED WARRANTIES

ANY IMPLIED WARRANTIES, INCLUDING IMPLIED WARRANTIES OF MERCHANTABILITY AND FITNESS FOR A PARTICULAR PURPOSE, SHALL BE LIMITED TO THE DURATION OF THE EXPRESS WARRANTIES SET FORTH ABOVE. IN SOME STATES LIMITATIOINS ON DURATION OF IMPLIED WARRANTIES DO NOT APPLY.

LIMITATION OF REMEDIES

IN NO EVENT SHALL PHILIPS OR ANY OF ITS AFFILIATED OR SUBSIDIARY COMPANIES BE LIABLE FOR ANY SPECIAL, INCIDENTAL OR CONSEQUENTIAL DAMAGES BASED UPON BREACH OF WARRANTY, BREACH OF CONTRACT, NEGLIGENCE, TORT, OR ANY OTHER LEGAL THEORY. SUCH DAMAGES INCLUDE, WITHOUT LIMITATION, LOSS OF SAVINGS OR REVENUE; LOSS OF PROFIT; LOSS OF USE; THE CLAIMS OF THIRD PARTIES INCLUDING, WITHOUT LIMITATION, DENTISTS AND DENTAL HYGIENISTS; AND COST OF ANY SUBSTITUTE EQUIPMENT OR SERVICES. SOME STATES DO NOT ALLOW THE EXCLUSION OR LIMITATION OF INCIDENTAL OR CONSEQUENTIAL DAMAGES.

www.philips.com/Sonicare

©2014 Koninklijke Philips N.V. (KPNV). All rights reserved.
Philips and the Philips shield are trademarks of KPNV.
AirFloss, Sonicare and the Sonicare logo are trademarks
of Philips Oral Healthcare, LCC. and/or KPNV.

100% recycled paper

4235.020.4312.1
